# Efficient Generation of Plasmacytoid Dendritic Cell from Common Lymphoid Progenitors by Flt3 Ligand

**DOI:** 10.1371/journal.pone.0135217

**Published:** 2015-08-11

**Authors:** Yi-Ling Chen, Shiun Chang, Ting-Ting Chen, Chien-Kuo Lee

**Affiliations:** Graduate Institute of Immunology, National Taiwan University College of Medicine, Taipei, Taiwan; Harvard Medical School, UNITED STATES

## Abstract

Dendritic cells (DCs), including conventional DCs (cDCs) and plasmacytoid DCs (pDCs) are critical for initiating and controlling the immune response. However, study of DC, particularly pDC, function is hampered by their low frequency in lymphoid organs, and existing methods for in vitro DC generation preferentially favor the production of cDCs over pDCs. Here, we demonstrated that pDCs could be efficiently generated in vitro from common lymphoid progenitors (CLPs) using Flt3 ligand (FL) in three different culture systems, namely feeder-free, BM-feeder and AC-6-feeder. This was in stark contrast to common DC progenitors (CDPs), in which cDCs were prominently generated under the same conditions. Moreover, the efficiency and function of pDCs generated from these three systems varied. While AC-6 system showed the greatest ability to support pDC development from CLPs, BM-feeder system was able to develop pDCs with better functionality. pDCs could also be expanded in vivo using hydrodynamic gene transfer of FL, which was further enhanced by the combined treatment of FL and IFN-α. Interestingly, IFN-α selectively promoted the proliferation of CLPs and not CDPs, which might contribute to enhanced pDC development. Together, we have defined conditions for in vitro and in vivo generation of pDCs, which may be useful for investigating the biology of pDCs.

## Introduction

Plasmacytoid DC (pDCs) are innate immune cells that are capable of producing large quantities of type I interferon (IFN-I) upon stimulation and activation [1, 2]. The frequencies of pDCs are low and their life span is short compared to other immune cells. Therefore, they are constantly replenished from their progenitors to maintain their homeostasis at steady state [3]. Progenitors of both myeloid and lymphoid lineages are able to generate pDCs [4, 5]. Recently, two newly identified progenitors with immunophenotypes of lin^-^CD11c^-^BST2^+^IL-7Rα^+^ and lin^-^c-Kit^int/lo^Flt3^+^M-CSFR^-^, respectively, were reported to show a greater pDC potential [6, 7]. In fact, we and others also showed that CLPs had the ability to generate more pDCs than cDCs [6, 8]. Nevertheless, the developmental origins of pDCs are still not completely understood.

While several cytokines, such as Flt3 ligand (FL), M-CSF, and GM-CSF have been shown to regulate pDC development, the most physiologically relevant cytokine for maintaining the homeostasis of pDCs at steady state is FL [5, 9, 10]. DCs, including pDCs, are generated from Flt3-expressing progenitors, regardless of lymphoid or myeloid lineage [11, 12]. Targeted deletion of FL or Flt3 in mice results in severely impaired production of pDCs in addition to cDCs and interstitial dermal DCs [13, 14].

Several transcriptional factors are involved in regulation of pDC development. E2-2/*Tcf4* is predominantly expressed in human and mouse pDCs. Genetic ablation of E2-2 results in reduction of pDCs but not of other lineages of DCs [15]. E2-2 directly activates multiple pDC-enriched genes, including transcription factors involved in pDC development, like *Spib*, *Irf8* and function, such as *Irf7* [16, 17]. Therefore, E2-2 appears to be an essential and specific transcription factor for pDC development. Moreover, continuous expression of E2-2 is also required to maintain the lineage identity of mature pDCs. Inducible deletion of E2-2 in mature pDCs results in conversion of pDCs into cells with cDC properties, including loss of pDC markers, acquisition of dendrites, induction of cDC signature genes and increase of cDC functions [17].

pDCs express TLR7 and TLR9 and are capable of producing large amounts of IFN-I and upregulating MHC class II and costimulatory molecules such as CD80 and CD86 in response to viral infections or TLR stimulation [18, 19]. pDC ablation using BDCA2-DTR transgenic mice reveals that pDCs are essential for early IFN-I production and influence the accumulation of viral specific NK and CD8^+^ T cells in a virus-dependent manner [20]. Mouse pDCs also selectively express BST2/PDCA1 and Siglec-H. A deficiency of Siglec-H in pDC results in enhanced production of IFN-I and proinflammatory cytokines in response to CpG ODN, suggesting that Siglec-H negatively regulates pDC functions [21]. Interestingly, conditional ablation of pDCs using Siglec-H-DTR transgenic mice enhances the activity of antigen specific CD4 but reduces the activity of CD8, the numbers of CD4^+^FoxP3^+^ Treg cells and the host defense against bacterial infections in vivo, suggesting that pDCs also play roles in initiation of the adaptive immune responses [21].

Cytokines have been shown to facilitate DCs generation in vitro from a number of progenitors [22, 23]. Among them, GM-CSF and FL, respectively, are the most commonly used cytokines. While GM-CSF preferentially induces cDC formation, FL can promote the development of both cDCs and pDCs. Cotreatment of GM-CSF and FL inhibits FL-dependent pDC development while cDC generation is not affected [24]. GM-CSF selectively utilizes its signal mediator STAT5 to bind the promoter and suppress *Irf8* and other pDC specific transcription factors in lin^-^Flt3^+^ progenitors, which impedes pDC generation. In vivo delivery of FL either by subcutaneous injection of the cytokine or hydrodynamic injection of the FL-expressing plasmid can also enhance pDCs production [8, 12, 25].

Although FL can induce pDC generation from BM, CDPs, CMPs and other progenitors, the process was less efficient compared to cDC generation [11, 26–28]. Here we have demonstrated that FL can robustly expand pDCs in vitro from CLPs using three different culture systems and in vivo using HGT of FL in combination with IFN-α. The functions of the pDCs were also evaluated. These results may provide crucial information for further characterization of the developmental process and functionality of pDCs.

## Materials and Methods

### Ethics Statement

C57BL/6 wild-type mice were purchased from the National Laboratory Animal Center (NLAC), Taiwan. CD45.1xCD45.2 congenic mice were bred at the Laboratory Animal Center of National Taiwan University College of Medicine, Taiwan. All mice were bred and kept under specific pathogen-free conditions. Protocol and the use of the animals were reviewed and approved by the Institutional Animal Care and Use Committee of National Taiwan University College of Medicine (Permit Number: 20120075). Water and food were provided sufficiently daily and all efforts were made to minimize suffering of the animals.

### Antibodies and flow cytometry

Staining was performed with hybridoma supernatant 2.4G2 Fc block (ATCC HB197) in FACS staining buffer at 4°C. The following antibodies were purchased from Biolegend: biotin-anti-CD11c (N418), Brilliant Violet 650 anti-CD11c (N418), PE-anti-CD11b (M1/70), PE-anti-CD3 (17A2), PE-anti-CD8 (53–6.7), PE-anti-Gr-1 (RB6-8C5), PE-anti-CD19 (eBio1D3), PE-anti-TER119 (TER-119), PE-anti-Thy1.1 (HIS51), PE-anti-NK1.1 (PK136), APC-anti-B220 (RA3-6B2), FITC anti-TLR9 (M9.D6), Brilliant Violet 421 anti-B220 (RA3-6B2), PerCP-eFluor710-anti-Siglec-H (eBio440c), PerCP-eFluor710-anti-c-Kit (2B8), APC-anti-M-CSFR (AFS98), PE/Cy7-anti-IL-7Rα (A7R34), FITC-anti-Sca-1 (D7). PE-anti-MHCII (NIMR-4) and FITC-anti-MHCII (NIMR-4) were purchased from eBioscience, and FITC-anti-IFNα (RMMA-1) was purchased from PBL Assay Science. APC-Cy7-Streptavidin was used as the secondary antibody (Biolegend). Flow data were analyzed with FlowJo (FLOWJO LLC) software.

### Progenitor analysis and cell sorting

BM cells following lysis of RBC were stained with PE-conjugated lineage markers, including anti-CD3, anti-CD8, anti-B220, anti-CD19, anti-CD11b, anti-Gr-1, anti-Thy1.1, anti-NK1.1, anti-TER119, and anti-MHC-II. CLPs and CDPs defined as lin^-^c-kit^int^Sca-1^int^M-CSFR^-^IL-7Rα^+^ [29] and lin^-^c-kit^int^Flt3^+^M-CSFR^+^IL-7Rα^-^ [28], respectively, were sorted with BD FACSAria IIIu (BD Biosciences).

### In vitro DC development

For feeder-free (FF) system, 1x10^4^ CLPs were seeded in 96-well U-bottom plates supplied with 100 ng/ml of hFL-Ig (FL in short, produced in-house from an expression cell line in July 2006 provided by M. Manz, University Hospital Zürich, Zürich, Switzerland [30]), in 80 μl culture medium for 6 d. For BM-feeder (BF) system, 1x10^4^ CLPs (CD45.2) were cocultured with 3.5 x 10^5^ total bone marrow cells of WT CD45.1xCD45.2 congenic mice supplied with 100 ng/ml FL in 350 μl culture medium in non-tissue culture-treated 24-well plates for 6 d. Equal volume of fresh medium (80 μl for FF system and 350 μl for BF system) containing 100 ng/ml FL was added every 3 d. For AC-6.21 (AC-6 in short, in Sep. 2009 provided by I. Weissman, Stanford University, Stanford, CA, [31]) feeder system, AC-6 were first γ-irradiated with 3000 rad (30 Gy). Unless otherwise indicated, the irradiated cells were seeded at 5.9x10^4^/well into 12-well plates one day earlier. Sorted CLPs at 500 cells/well were cocultured with the AC-6, which would just reach confluency, supplied with the indicated doses of FL for 16–20 d. Progeny cells from BF system were stained with antibodies to CD45.1 and CD45.2 in addition to antibodies against CD11c, CD11b and B220, and analyzed by flow cytometry. Progeny cells from FF and AC-6 systems were stained with antibodies to CD11c, CD11b, and B220. CDCs and pDCs are CD11c^+^CD11b^+^B220^-^ and CD11c^+^CD11b^-^B220^+^, respectively.

### Hydrodynamic gene transfer (HGT)

HGT was done as described [8]. Mice were intravenously injected with 10% body-weight sterile dPBS containing plasmids within 5 seconds. For FL treatment, mice were injected with 5 μg pcDNA3-mFL (provided by T.-C. Wu at Johns Hopkins University, Baltimore, MD). For FL and IFN-α treatment, mice were injected with 2.5μg of pcDNA3-mFL and 0.25 μg pcDNA3.1-mIFN-α4 (provided by L.-H. Hwang at National Yang-Ming University, Taipei, Taiwan).

### Intracellular staining for IFN-α

Intracellular staining procedure was performed according to the instructions of BD Cytofix/Cytoperm kits. For intracellular TLR9 staining, pDCs were fixed and permeabilized overnight and stained with FITC-anti-TLR9 antibodies in BD Perm/Wash buffer. For intracellular IFN-α staining, sorted pDCs were stimulated with CpG ODN (CpG 1826, Sigma) in vitro for 20 h. Monensin 2μM (eBioscience) was added into the culture for another 4 h prior to fixation. The treated cells were fixed, permeabilized and stained in BD Perm/Wash buffer using FITC-anti-mouse IFN-α antibody.

### Virus infection

DCs derived from CLPs using AC-6-feeder system for 16 d were stained with anti-CD11b-PE first, followed by negative selection for pDCs using EasySep PE-selection kit (Stemcell). Typically, the purity of pDCs after negative selection was between 92–95%. Purified pDCs at 1.2x10^5^ were infected with or without VSV at an MOI of 10 for 18 h. The culture supernatant were collected and subjected to ELISA for IFN-α (PBL Assay Science) as described in the following.

### Quantitative RT-PCR

The mRNA of cDCs and pDCs was prepared using TurboCapture kit (QIAGEN). The cDNA was synthesized with HiScript I reverse transcriptase (BIONOVAS) and then subjected to RT-qPCR using primers to *Tcf4*, *Rag1*, *Id2*, *Ifna*, *Ifnb*, *Actb* and/or *Rpl7*. Data were normalized to *Actb*. Primer sequences are in the Table 1.

**Table 1:** Primer sequences for quantitative PCR.

Gene	Forward	Reverse
*Tcf4*	5’-AGAAGGAACGGAGGATGG-3’	5’-CTTGTCGCTCTTCAGGTG-3’
*Rag1*	5’-GGCTAGGGTCAGCAGCAAGGA-3’	5’-CACGGGATCAGCCAGAATGTGTTC-3’
*Id2*	5’-AACATGAACGACTGCTACTC-3’	5’-CTGACGATAGTGGGATGC-3’
*Ifna*	5'-CTTCCACAGGATCACTGTGTACCT-3'	5'- TTCTGCTCTGACCACCTCCC-3'
*Ifnb*	5'-ATGAGTGGTGGTTGCAGGC-3'	5'- TGACCTTTCAAATGCAGTAGATTCA-3'
*Rpl7*	5’-TCAACAAGGCTTCAATTAACAT-3’	5’-CAATCAAGGAATTATCTGTCAA-3’
*Actb*	5’-AGGTGTGCACCTTTTATTGGTCTCAA-3’	5’-TGTATGAAGATTTGGTCTCCCT-3’

### Proliferation assay

For proliferation assay, BrdU (0.8 mg/mouse, Sigma) was intraperitoneally injected into mice that were in vivo delivered with the indicated plasmids using HGT, at final 2 h before CLPs or CDPs were sorted, fixed and subjected to intracellular staining using anti-BrdU antibody (e-Bioscience) as described [32].

### ELISA and bioassay for cytokine detection

For FL and IFN-α detection, a mouse FL DuoSet ELISA kit (R&D Systems) and a mouse IFN-α ELISA kit (PBL Assay Science), respectively, were used. The ELISA procedure was conducted according the manufacturer’s instruction. For IFN-I detection, STAT3KO MEFs [33] stably transfected with ISRE-RFP reporter plasmid (generated in-house) were seeded at 7x10^3^/well into 96-well plates one day earlier. Serial dilution of serum or culture supernatant from the treated mice or cells was added to the reporter cell line. Forty eight hours later, cells were harvested and subjected to flow cytometry and RFP^+^ cells were analyzed. The production of IFN-I was calculated according to a standard of IFN-α4 treatment in the same cell line.

### Statistical analyses

All values are shown as mean ± SD. Two-tailed, unpaired *Student’s t* test was used to assess statistical significance: *, P < 0.05; **, P < 0.01; and ***, P < 0.005.

## Results

### In vitro expansion of pDCs from CLPs

**Fig 1:**
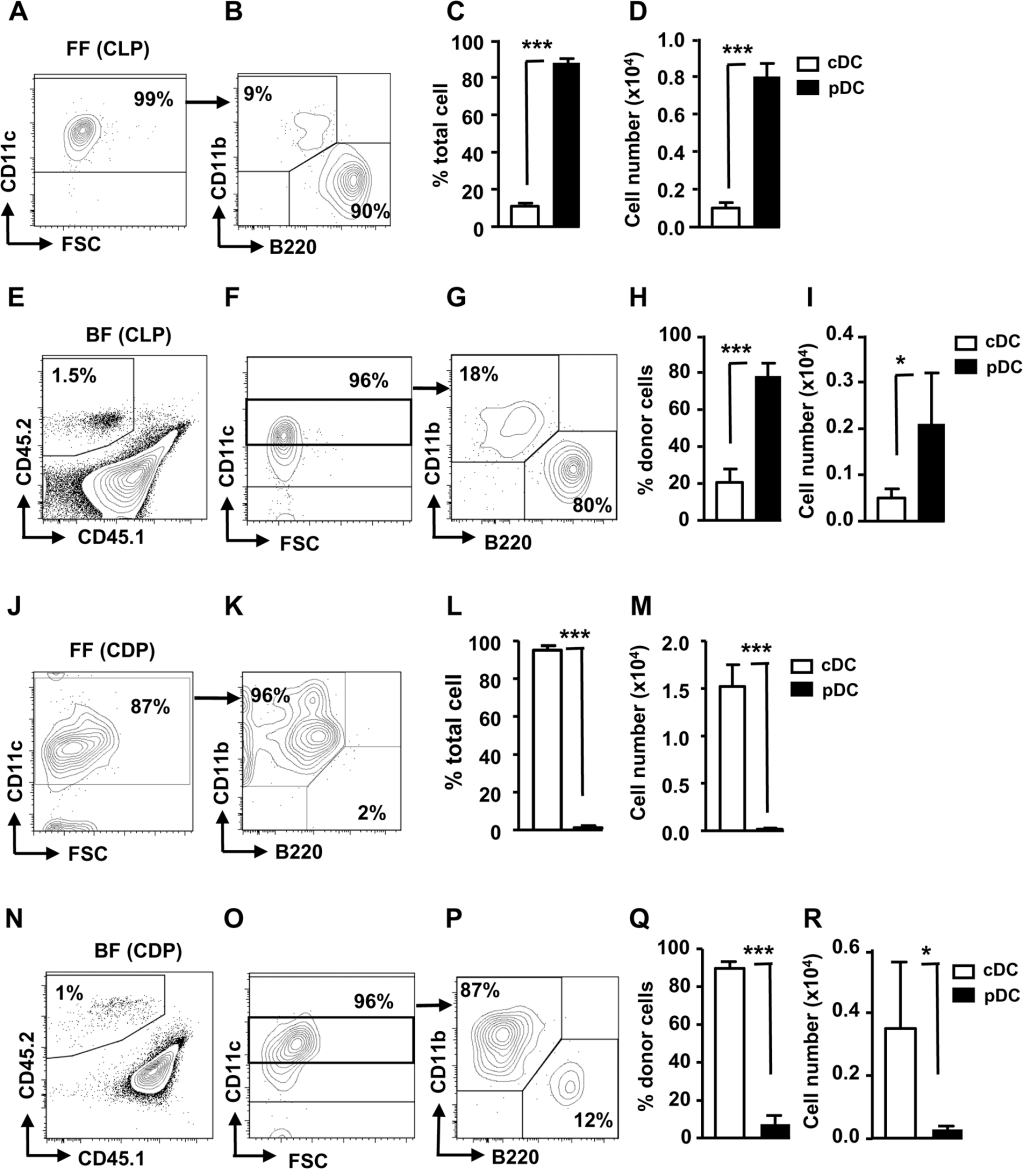
In vitro development of pDCs from CLPs. (A-D) CD45.2^+^ CLPs (1x10^4^) were cultured alone (feeder-free or FF, n = 3) or (E-I) cocultured with CD45.1xCD45.2 (2x10^5^) BM cells (BM-feeder or BF, n = 3–5) in the presence of 100 ng/ml FL for 6 d. (J-M) Same as in (A-I), except CD45.2^+^ CDPs were used. The progeny cells were stained with antibodies to CD11c, CD11b, and B220 and analyzed for cDCs (CD11c^+^CD11b^+^B220^-^) and pDCs (CD11c^+^CD11b^-^B220^+^) by flow cytometry. Mean percentages and cell numbers of cDCs and pDCs are shown. *, p<0.05, ***, p<0.005.

We have previously shown that CLPs could preferentially develop pDC in vitro [8]. Therefore, we further investigated pDC potential of CLPs. CLPs were first sorted out from BM of WT mice (S1 Fig), and then cultured in vitro using three different systems, namely feeder-free (FF), BM-feeder (BF) and AC-6-feeder (AC-6). In FF system, the majority of DCs developed were pDCs, while relatively few cDCs were generated (Fig 1A–1D). Likewise, pDCs were still mainly developed in BF system (Fig 1E–1H). We also sorted out CDPs and did in vitro culture using FF and BF systems. In contrast to CLPs, CDPs predominantly generated cDCs in FF (Fig 1J–1M) and BF (Fig 1N–1Q) systems. Recently, a clonogenic progenitor (lin^-^c-Kit^int^/^lo^Flt3^+^M-CSFR^-^) with prominent pDC potential has been identified [7]. We also examined their pDC potential in FF system. Although they generated higher percentages of pDCs than did CDPs, their pDC potential was still lower than those of CLPs (S2 Fig). These results suggest that CLPs develop more pDCs than cDCs in vitro and that they have a greater pDC potential than do CDPs and M-CSFR^-^ DC progenitors.

### Dose-dependent effect of AC-6 feeder and FL on pDC development from CLPs

**Fig 2:**
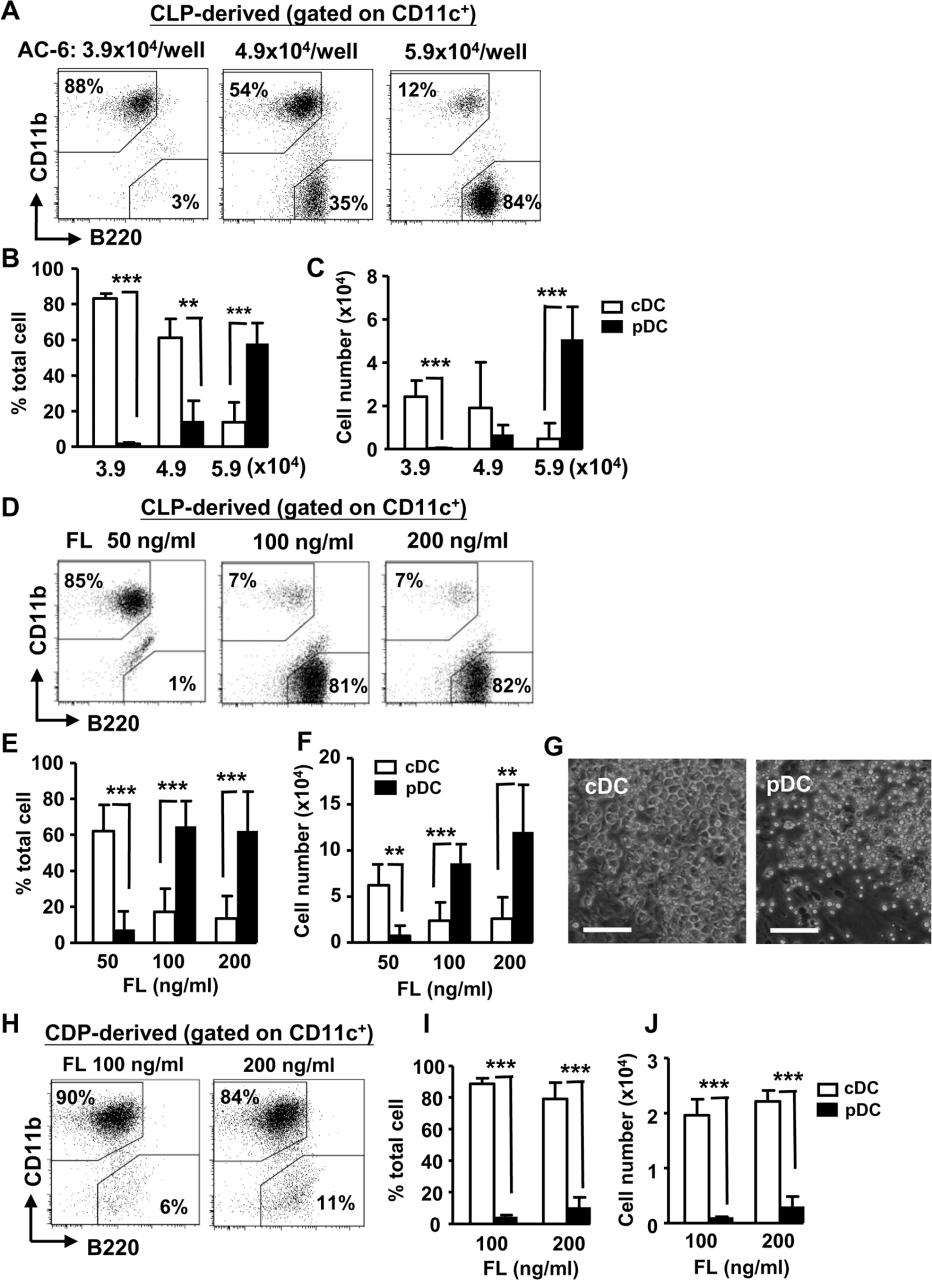
Dose-dependent effects of AC-6 feeder cells or FL on DC development from CLPs. (A) Sorted CLPs (5x10^2^ cells) were cocultured with the indicated numbers of AC-6 in the presence of FL (100 ng/ml) for 20 d. The progeny cells were stained with antibodies to CD11c, CD11b, and B220, gated on CD11c^+^ and analyzed for cDCs (CD11c^+^CD11b^+^B220^-^) and pDCs (CD11c^+^CD11b^-^B220^+^) by flow cytometry. Mean percentages (B) and cell numbers (C) of cDCs and pDCs are shown (n = 3–7). (D) Same as in (A), except CLPs were cocultured with 5.9x10^4^ AC-6 in the presence of the indicated doses of FL. Mean percentages (E) and cell numbers (F) of cDCs and pDCs are shown (n = 5–15). (G) Images of light microscopy of AC-6-derived cDC and pDC colonies are shown (scale bar 50 μm). (H) Same as in (D-F), except CDPs were cocultured with 5.9x10^4^ AC-6 in the presence of the indicated doses of FL for 12 d. Mean percentages (I) and cell numbers (J) of cDCs and pDCs are shown (n = 3). **, p<0.01, ***, p<0.005.

We next examined AC-6 system. To titrate the effect of AC-6, 500 CLPs were cultured with increased numbers of AC-6 in the presence of 100 ng/ml FL. Interestingly, AC-6 regulated pDC developmental potential in a dose-dependent manner. While lower numbers of AC-6 (3.9x10^4^/well) promoted cDCs development, higher numbers of AC-6 (5.9x10^4^/well) supported pDCs formation, resulting in increased pDC frequency in the culture (Fig 2A–2C). Moreover, higher numbers of AC-6 were also capable of supporting more pDC generation (Fig 2C). Under this condition, pDCs could be expanded as high as 100-fold. We next titrated dose effect of FL on DC development from CLPs using the optimal numbers of AC-6. Similar to the dose effect of AC-6 feeders, low-dose FL (50 ng/ml) preferentially promoted cDC development while high-dose FL (100–200 ng/ml) promoted pDC development (Fig 2D–2F). Moreover, the morphology of the colonies of pDCs and cDCs derived from AC-6 system were distinct; with cDCs larger and more adherent and pDCs smaller, rounded-shape and non-adherent (Fig 2G). However, the dose-dependent effect of FL on pDC development from CLPs was not observed in CDPs, as cDCs were still preferentially generated even at high-dose of FL (Fig 2H–2J). Taken together, while all three culture systems were able to generate pDCs from CLPs, AC-6 system had the greatest ability to generate pDCs. Moreover, there is dose-dependent effect of FL or AC-6 cells on pDC development from CLPs, at least, in AC-6 culture system.

### Activation and function of in vitro generated pDCs

**Fig 3:**
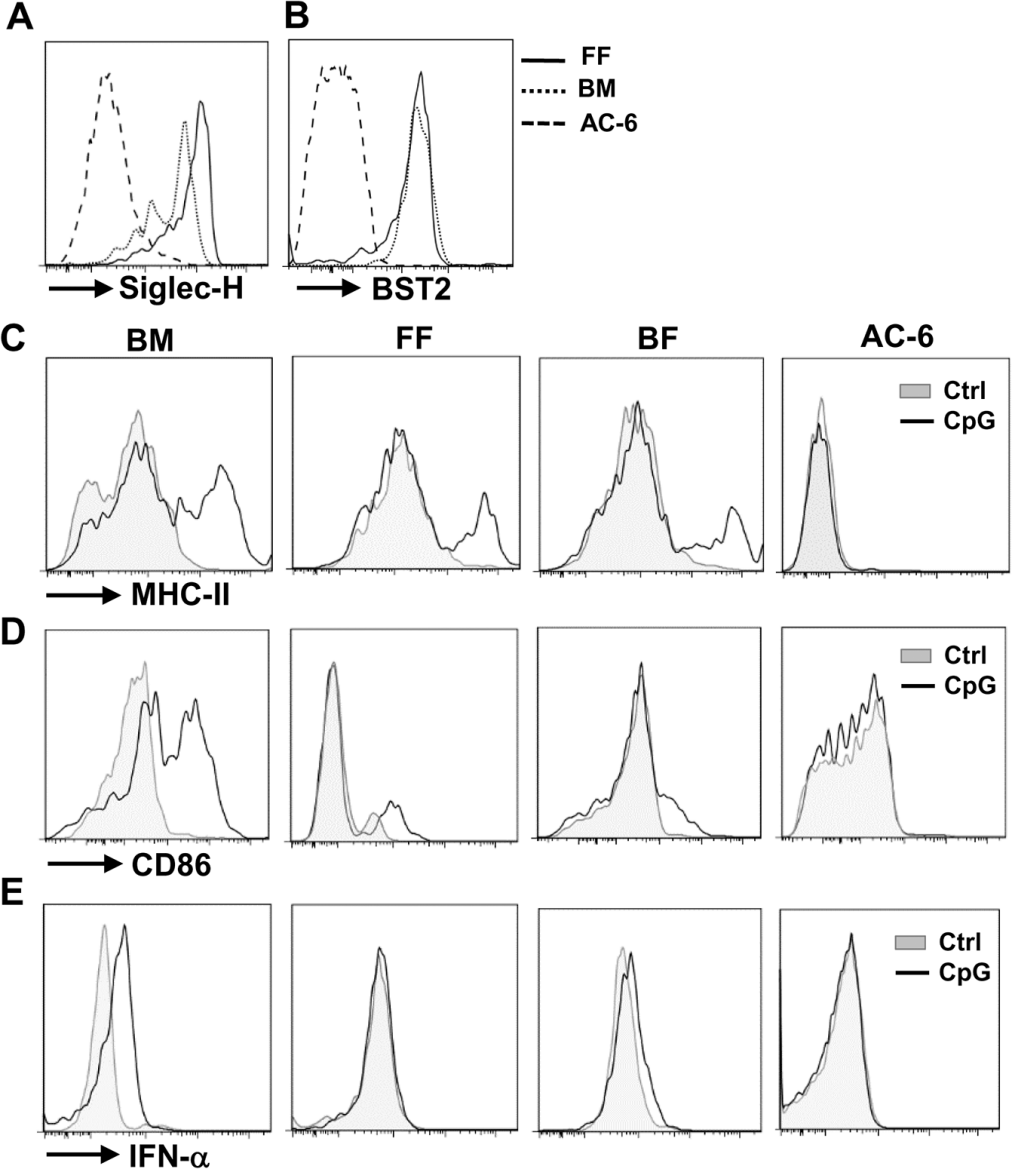
Maturation and function of in vitro generated pDCs. Surface expressions of Siglec-H (A) and BST2 (B) on pDCs derived from CLPs using FF, BF and AC-6 systems were shown (n = 3). (C) pDCs sorted from BM or BF culture system or DCs derived from FF and AC-6 culture system were treated with or without CpG ODN (1 μg/ml) for 24 h. Surface staining of MHC class II (C) and CD86 (D) or intracellular staining of IFN-α (E) on sorted BM pDCs or BF-pDCs or gated pDCs from FF- or AC-6 system are shown. One representative experiment out of three.

**Fig 4:**
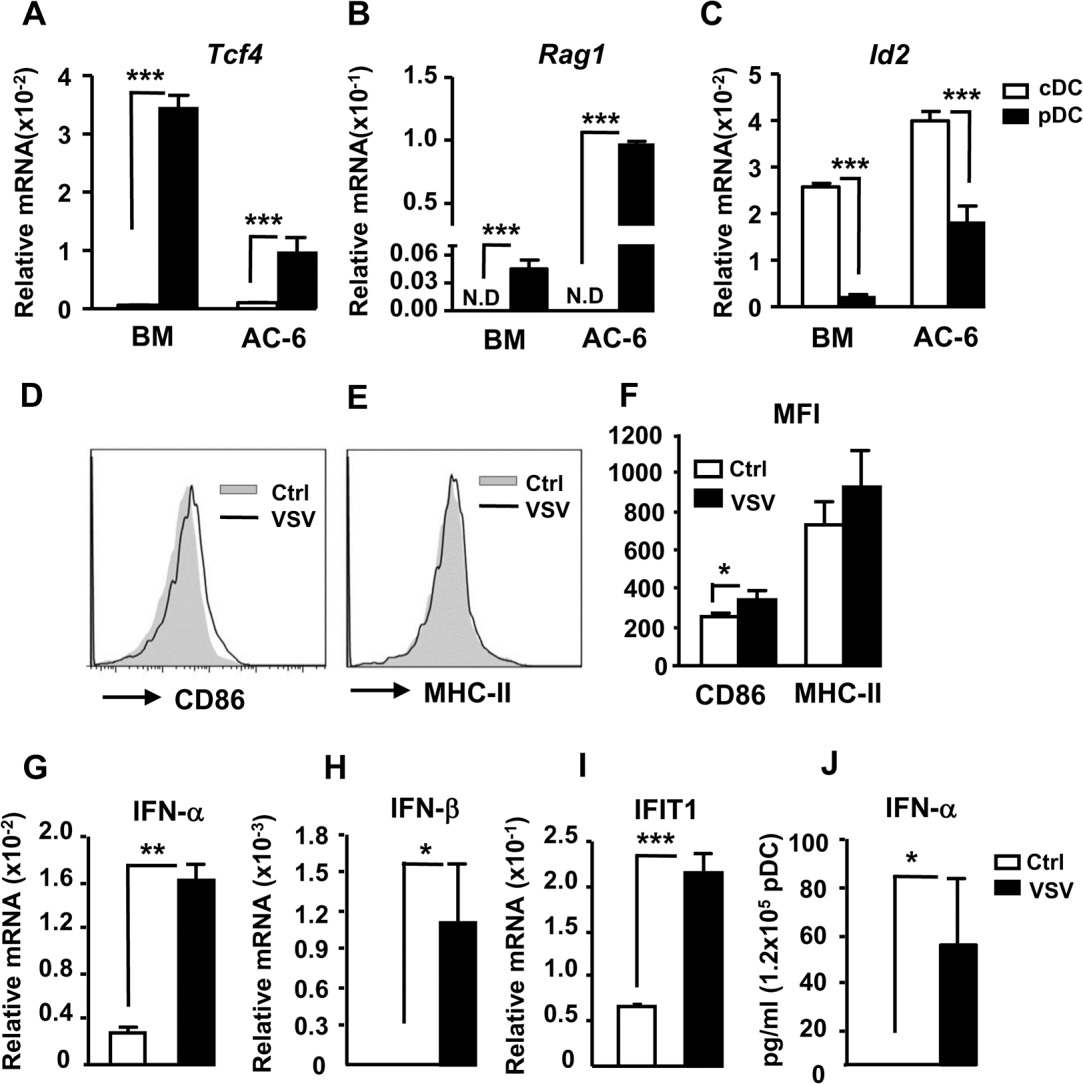
AC-6-pDCs highly express *Tcf4* and *Rag1* and produce IFN-I in response to viral infection. Total RNA prepared from sorted cDCs and pDCs from BM or AC-6 culture system were subjected to RT-qPCR using primers to *Tcf4* (A), *Rag1* (B) and *Id2* (C). Relative mRNA was normalized to *Actb*. Purified AC-6-pDCs (1.2x10^5^) were infected with or without EMCV or VSV at an MOI of 10 for 18h. The infected cells were stained with antibodies to CD11c, CD11b, B220, MHC class II and CD86 and analyzed by flow cytometry. Expression of CD86 (D) or MHC class II (E) on pDCs (CD11c^+^CD11b^-^B220^+^) is shown. (F) MFI of CD86 and MHC class II on AC-6-pDCs infected with or without VSV was shown. (n = 3) (G-J) Same as in (D-E), except the mRNA of the virus infected pDCs were prepared and subjected to RT-qPCR using primers to all *Ifnα* (G), *Ifnb* (H), *Ifit1* (I) and *Rpl7*. Relative mRNA was normalized to *Rpl7*, a reference gene. (J) Supernatant of the cells infected with virus was subjected to ELISA for IFN-α production. *, p<0.05, **, p<0.01 ***, p<0.005.

Since we have shown that CLPs exhibited a greater pDC potential in three different culture systems, we further characterize the phenotypes and functions of pDCs derived from CLPs. pDCs express unique surface markers, such as Siglec-H and BST2, and are the most potent IFN-I producing cells upon stimulation. Therefore, we first examined the expressions of these two markers on the pDCs derived from these three culture systems. While FF- and BF-derived pDCs (FF-pDCs and BF-pDCs, respectively) expressed Siglec-H and BST2, AC-6-derived pDCs (AC-6-pDCs) hardly expressed either molecule (Fig 3A and 3B). Moreover, basal levels of MHC class II on AC-6-pDCs were also lower than those on FF-pDCs or BF-pDCs (Fig 3C). CpG ODN stimulation upregulated MHC class II and CD86 on BM pDCs or FF- and BF-pDCs (Fig 3C and 3D). However, the expression of MHC class II and CD86 was not induced in AC-6-pDCs in response to CpG ODN. We next examined CpG-induced IFN-I in the pDCs by intracellular staining. While BM pDCs produced significant amount of IFN-α, BF-pDCs produced relatively low amounts. However, both FF-pDCs and AC-6-pDCs failed to produce IFN-α upon stimulation (Fig 3E). The unresponsiveness of AC-6-pDCs to CpG stimulation was not due to the lack of TLR9 expression because they appeared to express comparable levels of TLR9 compared to FF-pDCs (S3 Fig). Nevertheless, AC-6-pDCs, like BM pDCs, expressed higher levels of *Tcf4* (encodes E2-2) and *Rag1*, two pDC-specific factors, but lower levels of *Id2*, a cDC-specific factor, as compared to AC-6-cDCs and BM cDCs, respectively (Fig 4A–4C). Since TLR9 is expressed inside endosomes, we reasoned that the unresponsivenesse of AC-6-pDCs to CpG might be due to impaired ability to engulf the ligand. Therefore, we next infected AC-6-pDCs with vesicular stomatitis virus (VSV). Infection of VSV was able to upregulate small but significant amount of CD86 on AC-6-pDCs (Fig 4D) although there was no detectable change on MHC class II expression (Fig 4E). Moreover, VSV also induced the expression of IFN-α (Fig 4F), IFN-β (Fig 4G), IFIT1 (Fig 4H), an IFN-stimulated gene, and the production of IFN-α protein (Fig 4I) in AC-6-pDCs. We also infected BM-derived and CLP-derived pDCs from FF system with VSV and assessed the upregulation of CD86 and MHC class II and IFN-α-producing ability. Interestingly, basal and induced levels of CD86 on CLP-derived pDCs and cDCs were lower than those of BM-derived pDCs and cDCs (S4 Fig C, E, J, and L). Like AC-6-pDCs, MHC class II on CLP-derived pDCs was not induced by VSV infection (Fig 4E and S4K and S4M Fig). Moreover, CLP-derived pDCs produced significantly lower amounts of IFN-α than did BM-derived pDCs (S4 Fig G and N). Since CLP-derived pDCs from FF system and AC-6 system (Fig 4) produced similar levels of IFN-α, these results suggest that low IFN-I-producing ability of CLP-derived pDCs is probably an intrinsic property of CLP origin and not due to AC-6 culture system. Together, these results suggest that pDCs from BM- or FF- system display more mature phenotype and higher functionality than do pDCs from AC-6 system.

### IFN-I enhances FL-dependent pDC generation in vivo

**Fig 5:**
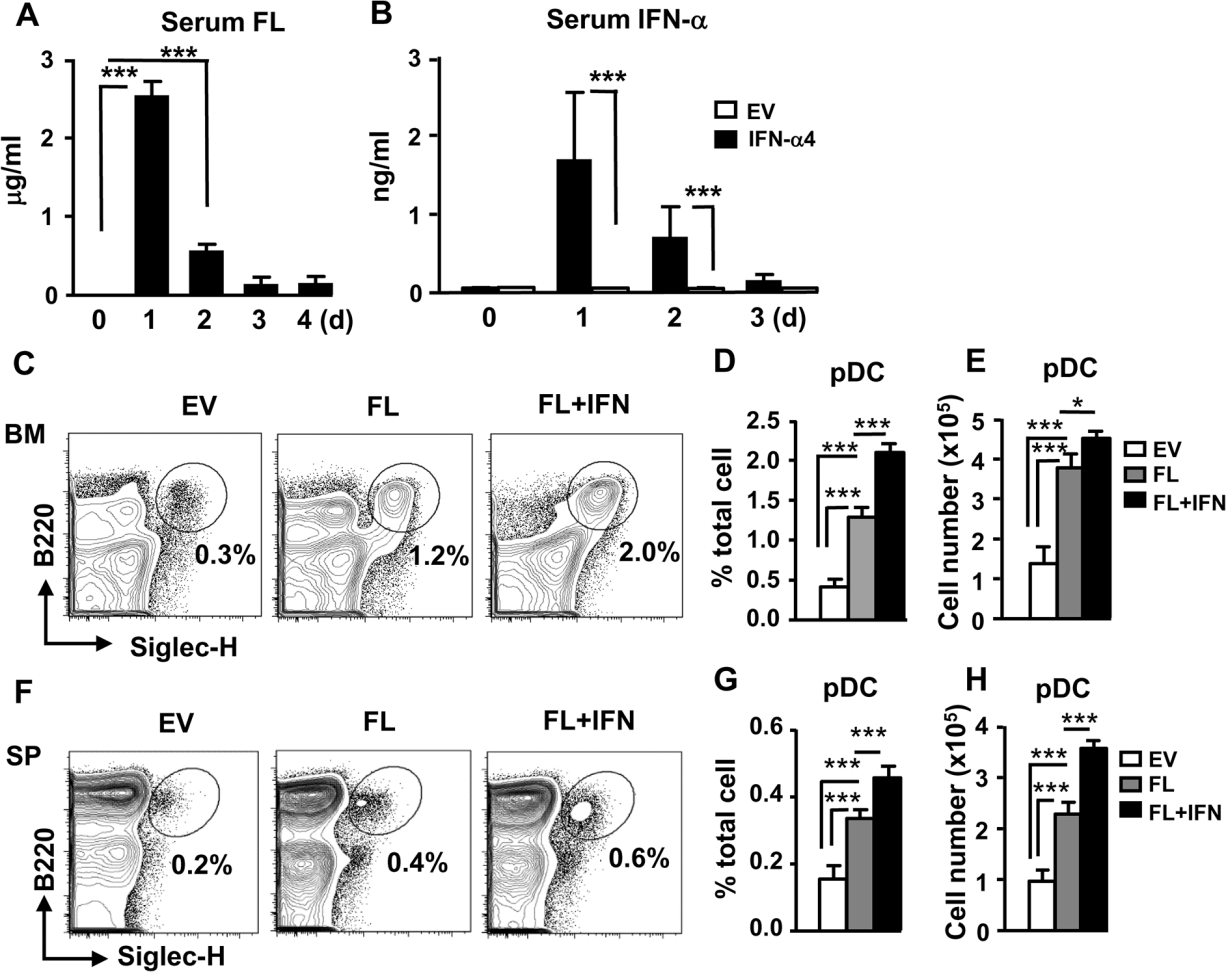
IFN-α enhances FL-dependent pDC development in vivo. Plasmid expressing FL (2.5 μg) or IFN-α4 (0.25 μg) was delivered into WT mice using HGT method. Serum FL (A) and IFN-α (B) was measured by ELISA. (n = 3) (C-H) Same as in (A-B), except empty vector (EV) or plasmid expressing FL (2.5 μg) or FL (2.5 μg) + IFN-α4 (0.25 μg) were delivered in vivo for 6 d. BM (C-E) and spleen cells (F-H) of the treated mice were stained, gated on CD11c^int^CD11b^-^, and analyzed for pDCs (CD11c^int^CD11b^-^B220^+^Siglec-H^+^). Mean percentages and cell numbers of pDCs following the treatments are shown (n = 3). *, p<0.05, ***, p<0.005.

**Fig 6:**
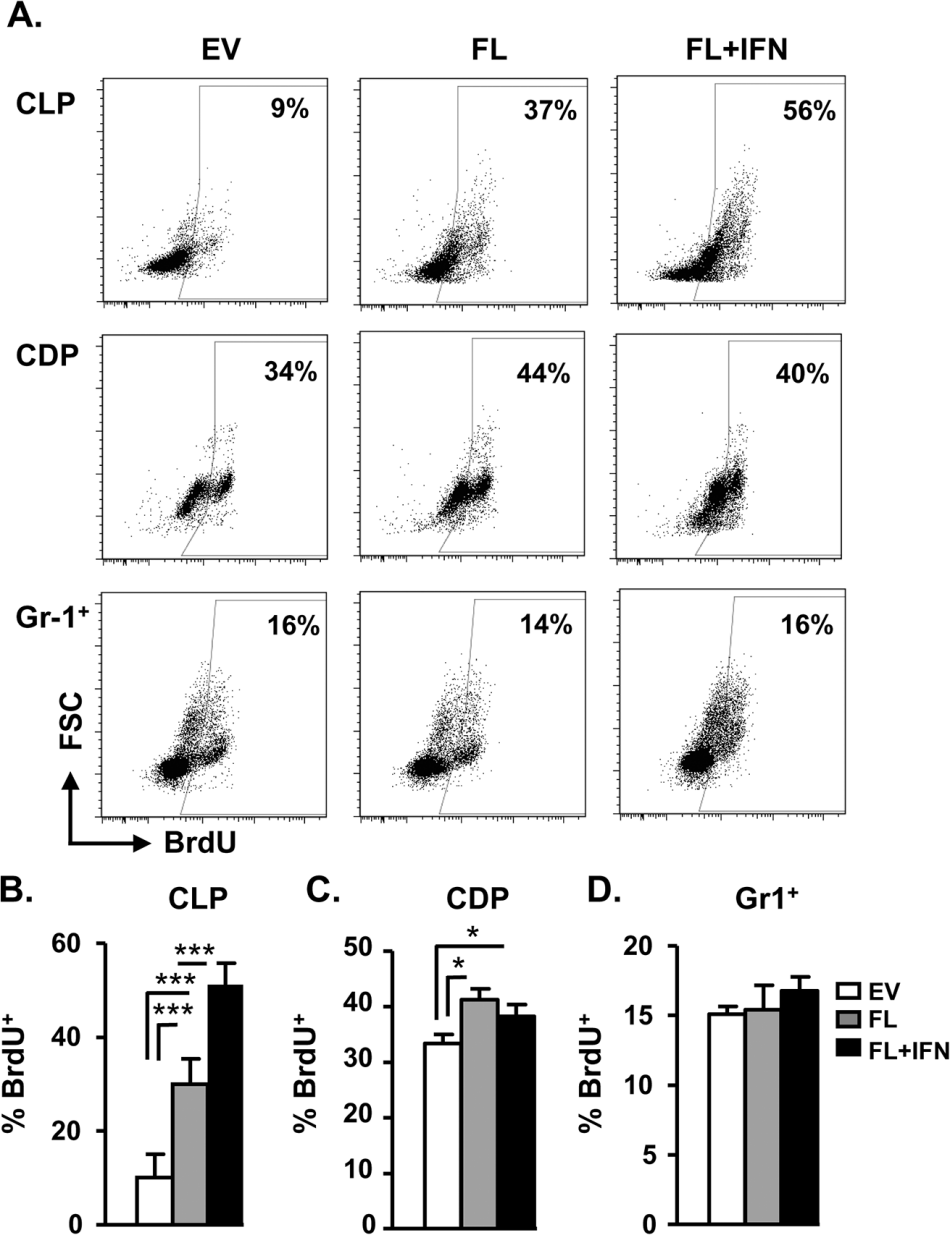
IFN-α enhances FL-dependent proliferation of CLPs, and not CDPs, in vivo. (A) Empty vector (2.5 μg), plasmid expressing FL (2.5 μg), or FL (2.5 μg) + IFN-α4 (0.25 μg) were delivered into WT mice using HGT method. The mice were then i.p. injected with 0.8 mg of BrdU at last 2 h. Twenty hours post HGT, CLPs (upper) or CDPs (middle) were sorted from the BM and subjected to intracellular staining for BrdU. Similarly, BM was stained and gated on Gr1^+^ (lower) cells before intracellular staining for BrdU. Mean percentages of BrdU^+^ cells in CLPs (B), CDPs (C) and Gr1^+^ cells (D) are shown (n = 3–4). *, p<0.05, ***, p<0.005.

We have previously demonstrated that IFN-I was capable of enhancing FL-dependent pDC production in vitro [8]. Here we delivered FL and IFN-I into mice and examined the enhancement of pDC expansion in vivo. The circulating levels of FL following hydrodynamic injection of FL-expressing plasmid rose to 2.5 μg/ml from 500 pg/ml and gradually subsided 3 d after the treatment (Fig 5A). Likewise, the production of serum IFN-α also significantly increased, at least, for 2 d following in vivo delivery of IFN-α4-expressing plasmid (Fig 5B). In vivo delivery of plasmid expressing FL alone increased the production of pDCs in the BM and spleen. Delivery of both FL and IFN-α4 further enhanced the percentages and numbers of pDCs (Fig 5C–5H). The proliferation of CLPs, as indicated by BrdU incorporation, was increased in BM CLPs following FL treatment compared to EV control (Fig 6A). Interestingly, IFN-α further enhanced FL-dependent proliferation of CLPs (Fig 6A and 6B). Although FL also slightly increased the proliferative activity of CDPs compared to EV control, IFN-α did not show enhanced mitogenic activity on CDPs (Fig 6A and 6C). However, a comparable proliferative activity was observed in BM Gr-1^+^ granulocytes following the same treatments (Fig 6A and 6D). To further confirm the direct effect of IFN-I, we isolated CLPs and CDPs and stimulated them with FL alone or FL plus IFN-α in vitro. Consistent with the in vivo results, IFN-α augmented FL-dependent proliferation in CLPs and not in CDPs (S5 Fig). Collectively, these results suggest that there is a selective enhancement of CLP proliferation by IFN-α in vitro and in vivo.

**Fig 7:**
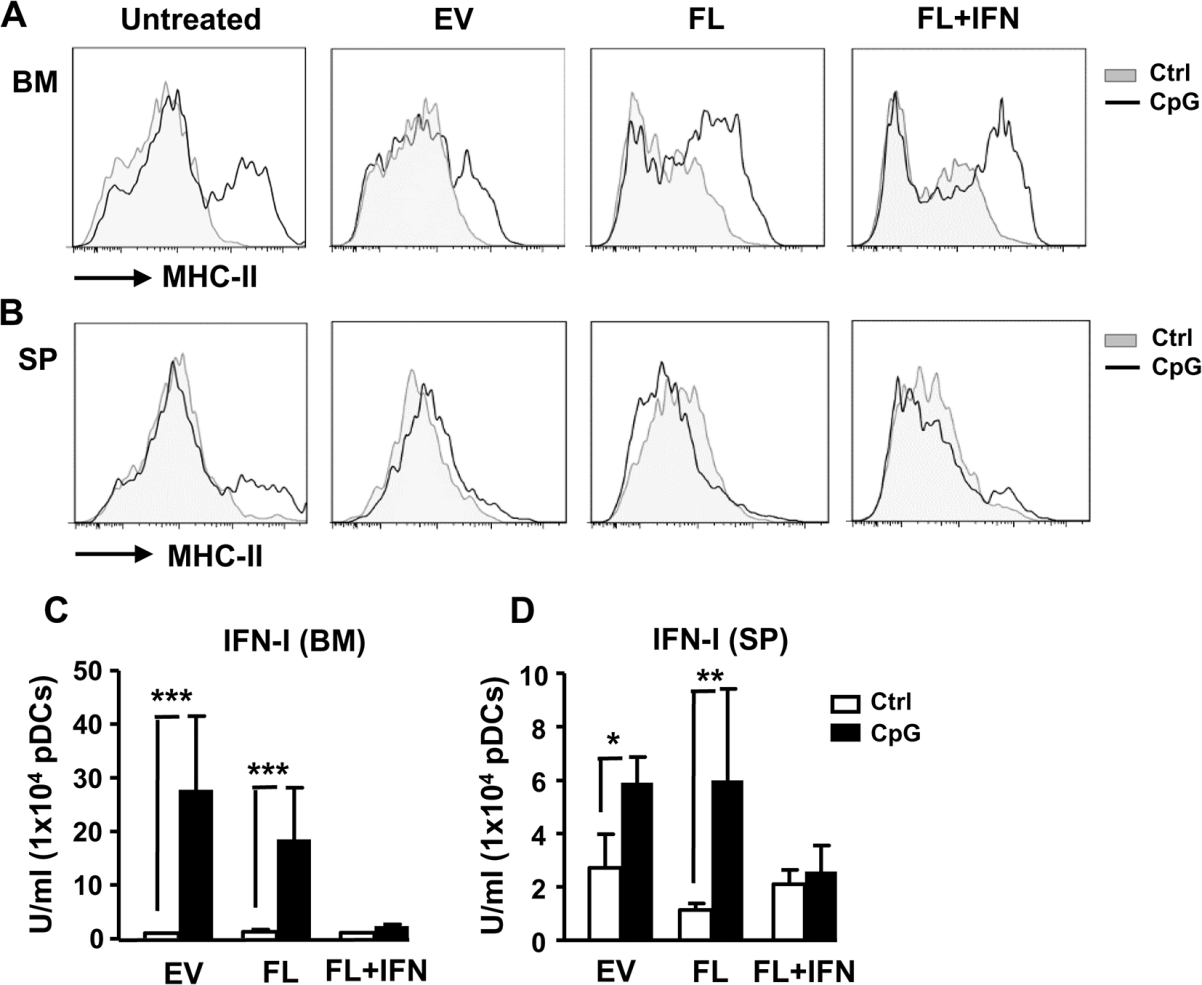
Activation and function of in vivo expanded pDCs. Empty vector (2.5 μg), plasmid expressing FL (2.5 μg), or FL (2.5 μg) + IFN-α4 (0.25 μg) was delivered in vivo into WT mice using HGT method for 6 d. pDCs sorted from the BM or spleen cells of the treated mice were stimulated with CpG ODN (1 μg/ml) for 24 h. (A-B) The expressions of MHC class II on the sorted BM (A) or spleen (B) pDCs with or without the stimulation were analyzed by flow cytometry. The production of IFN-α by the BM (C) or spleen (D) pDCs following the stimulation was measured by a bioassay as described in the Materials and Methods (n = 3). *, p<0.05, **, p<0.01, ***, p<0.005.

We next examined the functions of the in vivo expanded pDCs by sorting them out and stimulating them with CpG ODN. The expression of MHC class II was increased in BM pDCs of untreated or EV-treated mice in response to CpG (Fig 7A). The upregulation of MHC class II was also observed in BM pDCs of mice treated with FL or FL plus IFN-α4. However, the upregulation of MHC class II was less obvious in the treated splenic pDCs (Fig 7B). PDCs from mice treated with EV or FL were also able to produce significant amounts of IFN-I upon stimulation (Fig 7C and 7D). However, co-treatment of FL and IFN-α4 impeded IFN-I-producing ability in pDCs. Together, these results suggest that although IFN-I enhances pDC production, it may have adverse effects on IFN-I-producing ability in pDCs.

## Discussion

### Preferential pDC development from CLPs in vitro

While CLPs can develop both cDCs and pDCs in vitro, here we have defined the conditions to predominantly generate pDCs. Although three in vitro culture systems were able to promote pDC formation, their developmental efficiency and differentiation ability varied. Developmental efficiency, defined as the ratios of the number of pDCs generated over initial input number of CLPs, for FF and BF systems was 0.8 and 0.2 (Fig 1D and 1I), respectively, which was less efficient. In contrast, AC-6 system was able to robustly produce pDCs, in which one CLP could generate almost 100 pDCs under optimal conditions (Fig 2C). Differentiation ability, assessed by expression of pDC markers and responses to TLR9 ligand or virus infection, was better in FF and BF systems than in AC-6 system. It was interesting to note that AC-6-pDCs did not express Siglec-H or BST2. Their MHC class II or CD86 was not upregulated and they hardly produce IFN-I in response to CpG ODN despite that they expressed TLR9 (S3 Fig). Nevertheless, AC-6-pDCs expressed high levels of *Tcf4* and *Rag1* and low levels of *Id2* as opposed to AC-6-cDCs. Moreover, when AC-6-pDCs were infected with VSV, they were able to upregulate CD86 and express IFN-I and IFN-stimulated genes. One possibility for the differential response is that AC-6-pDCs may have a deficiency in CpG uptake because TLR9 is expressed in the endosomes. Viruses, on the other hand, were able to actively infect AC-6-pDCs, which permitted them to mount proper responses. Alternatively, viral infections might engage multiple pattern recognition receptors, thus triggering stronger signals that overcome the defect in AC-6-pDCs. Therefore, these results suggest that although AC-6 system has the greatest ability to support CLPs to develop pDCs, the AC-6-pDCs appeared to display only “pDC-like” phenotype due to lack of Siglec H/BST2 expression and TLR9 response.

AC-6 system has been used to facilitate DC development in vitro to dissect the roles of progenitors of lymphoid and myeloid lineages [12] and to define clonogenic property of CDPs and Lin^-^c-Kit^int/lo^Flt3^+^M-CSFR^-^, a newly identified DC progenitor [7, 28]. Moreover, we have previously used this system to reveal a role of IFN-I in FL-dependent pDC development from CLPs [8]. Therefore, it is a well-established system and it has provided invaluable information, at least, for the developmental control of DCs. Nevertheless, the functions of AC-6-DCs, particularly AC-6-pDCs, have not been characterized. Therefore, our findings have documented and raised the awareness of the inferior functionality of CLP-derived pDCs from AC-6 system. Therefore, we propose that while FF or BF system is suitable for characterization of immunophenotype and function of pDCs, AC-6 system is more appropriate for studying the homeostasis of pDCs.

### Feeder-free/ BM-feeder versus AC-6-feeder system

Although the exact mechanisms for the efficient DC generation from AC-6 system remain to be determined, cobblestone area-forming cells (CAFC) developed in AC-6 system might facilitate cell proliferation [34], which does not occur in FF or BF system. In addition to CAFC, hematopoietic cytokines secreted by the feeders and cell-cell contact between feeders and CLPs might also play a role. It is known that a stromal cell line can constitutively produce hematopoietic cytokines such as IL-7, SCF, M-CSF and FL to support hematopoiesis in vitro [35]. Moreover, heparan sulfate present on the surface of hematopoietic stromal cells has been shown to control adhesion and growth of hematopoietic stem and progenitor cells [36]. Therefore, differences in quantity or quality of these factors in FF, BF and AC-6 system may account for the distinct phenotypes. Although in vitro BM-derived pDCs using FF system are capable of secreting significant amounts of TNF/IL-6 and IFN-I in response to TLR7 or TLR9 stimulation [19, 26, 37], we and others showed that CLP-derived pDCs using BF, but not FF system, only secreted relatively limited amount of IFN-I upon CpG stimulation [6]. Therefore, in vitro derived pDCs from different precursors may show distinct properties.

### Dose-dependent effect of FL and AC-6 on DC development from CLPs

We have previous shown a dose-dependent effect of FL on DC development from CLPs [8]. Here we further demonstrated a similar effect for AC-6 on DC generation. At low numbers of AC-6, cDCs were preferentially developed, while at high numbers pDCs were predominantly generated. These results further support the model of instructive cytokine signals for lineage commitment [9, 38]. However, this phenomenon seemed to be limited to CLPs and not to CDPs, as CDPs mainly generated cDCs at low or high doses of FL. In fact, clonal analysis of pDC potential from CDPs using FL as the sole hematopoietic cytokine also reveals that pDC-restricted clones are only 1% in this population [6]. Interestingly, CLPs, and not CMP, are also the major pDC producers when using M-CSF to drive DC development in vitro [37]. Despite that FL or M-CSF alone preferentially induce pDC formation from CLPs, it should be noted that DC-poietin like FL, M-CSF and GM-CSF most likely function in concert to regulate DC homeostasis in vivo [39]. In fact, FL can act together with M-CSF to enhance the expansion of pDC from CDPs, suggesting that CDPs still have a potential to switch the developmental program for pDC formation [28].

### Effect of IFN-I on pDC development and functions

We have previously shown that FL induces IFN-I production in CLPs, which in turn upregulates Flt3 on CLPs and promotes survival, proliferation of CLPs and their differentiation into pDCs in vitro [8]. Here we extended these findings and showed that in vivo administration of FL and IFN-I together could further enhance pDC generation compared to single treatment of FL. IFN-I has been shown to stimulate proliferation of HSC in a STAT1- and Sca-1-dependent manner [40]. Here, we also found that IFN-α selectively enhanced FL-dependent proliferation of CLPs both in vitro and in vivo, while it had no effect on CDPs or Gr-1^+^ granulocytes. These results suggest that differentiation process may alter intracellular signaling networks in CLPs, CDPs, and granulocytes, respectively, resulting in distinct phenotypes upon IFN-I stimulation. Interestingly, in vivo IFN-I treatment also inhibited the production of IFN-I from pDCs in response to CpG. It has been reported that TGFβ and IL-10 block CpG- or influenza virus-induced IFN-I production in splenic pDCs, with TGFβ inhibiting proximal signaling via TLR9-induced IFN-I mRNA expression and IL-10 blocking IFN-I receptor-mediated autocrine signaling at a later stage [41]. Therefore, it is tempting to speculate that pretreatment of IFN-I and FL during pDC expansion induces the production of TGFβ or IL-10 which, in turn, attenuates IFN-I production from pDCs in response to CpG or viral infections.

In sum, we have defined several methods to efficiently expand pDCs in vitro and in vivo, which may facilitate the investigation of the developmental process and maybe the functions of pDCs. Moreover, these findings may provide a direction for therapeutic intervention for pDC-associated autoimmune diseases such as systemic lupus erythematosus (SLE) and psoriasis [42].

## Supporting Information

S1 Fig Gating strategies for CLPs and CDPs. CLPs were defined as lin^-^c-kit^int^ Sca-1^int^M-CSFR^-^IL7Ra^+^. CDPs were defined as lin^-^c-kit^int^ Flt3^+^M-CSFR^+^IL-7Ra^-^.
(TIF)

S2 Fig In vitro development of DCs from CLPs, CDPs and M-CSFR^-^ DC progenitors. (A) CLPs, CDPs, and M-CSFR^**-**^ DC progenitors (Lin^-^c-Kit^int/lo^Flt3^+^ M-CSFR^-^) were sorted and cultured in vitro under feeder-free conditions in the presence of FL (100 ng/ml) for 6 d. The progeny cells were stained, gated on CD11c^+^ and analyzed by flow cytometry. (B) Mean percentages of cDC (CD11c^+^CD11b^+^ B220^-^) and pDC (CD11c^+^CD11b^-^B220^+^) are shown (n = 4).
(TIF)

S3 Fig AC-6-pDCs express TLR9. pDCs sorted from BM or BF system or DCs derived from FF and AC-6 system were treated with or without CpG ODN (1 μg/ml) for 24 h. Expressions of TLR9 on sorted or gated pDCs are shown.
(TIF)

S4 Fig BM-derived pDCs from feeder-free system are better responders and produce higher levels of IFN-I than do CLP-derived pDCs in response to VSV infection. BM or CLPs of WT mice were cultured in vitro in the presence of FL 100 ng/ml for 6 d. BM (A-G) or CLP (H-N)-derived cells were stained with anti-CD11c, anti-CD11b, anti-B220 and anti-Siglec-H, gated on CD11c^+^ cells and analyzed for cDCs (CD11c^+^CD11b^+^B220^-^) and pDCs (CD11c^+^CD11b^-^B220^+^). The expression of Siglec-H on cDCs and pDCs from BM (B) or CLPs (I) is shown. Magnetic bead-purified pDCs from BM-derived DCs and CLP-derived DCs were infected with VSV at an MOI = 10 for 24 h. Infected pDCs were stained for anti-CD86 (C, E, J, L) and anti-MHC-II (D, F, K, M). (n = 3–4) The supernatant of the infected BM-derived pDCs (G) or CLP-derived pDCs (N) was subjected to ELISA for measuring secreted IFN-α. (n = 3), *P<0.05, **P<0.01, ***P<0.005.
(TIF)

S5 Fig IFN-α4 enhances the proliferation of CLPs in vitro. CLPs (A) and CDPs (B) sorted from WT mice were stimulated with or without FL alone (100 ng/ml) or FL (100 ng/ml) plus IFN-α4 (100 U/ml) for 8 h. BrdU (100 μg/ml) was added at the last 4 h before subjecting the treated cells to intracellular staining for BrdU as described in the Materials and Methods.
(TIF)

## References

[1] Gilliet M, Cao W, Liu YJ. Plasmacytoid dendritic cells: sensing nucleic acids in viral infection and autoimmune diseases. Nature reviews Immunology. 2008;8(8):594–606. Epub 2008/07/22. 10.1038/nri2358 . 18641647

[2] Reizis B, Bunin A, Ghosh HS, Lewis KL, Sisirak V. Plasmacytoid dendritic cells: recent progress and open questions. Annu Rev Immunol. 2011;29:163–83. Epub 2011/01/12. 10.1146/annurev-immunol-031210-101345 . 21219184 PMC4160806

[3] Merad M, Manz MG. Dendritic cell homeostasis. Blood. 2009;113(15):3418–27. Epub 2009/01/30. 10.1182/blood-2008-12-180646 19176316 PMC2668851

[4] Reizis B. Regulation of plasmacytoid dendritic cell development. Curr Opin Immunol. 2010;22(2):206–11. Epub 2010/02/11. 10.1016/j.coi.2010.01.005 20144853 PMC2854232

[5] Shortman K, Sathe P, Vremec D, Naik S, O'Keeffe M. Plasmacytoid dendritic cell development. Adv Immunol. 2013;120:105–26. Epub 2013/09/28. 10.1016/b978-0-12-417028-5.00004-1 . 24070382

[6] Sathe P, Vremec D, Wu L, Corcoran L, Shortman K. Convergent differentiation: myeloid and lymphoid pathways to murine plasmacytoid dendritic cells. Blood. 2013;121(1):11–9. Epub 2012/10/12. 10.1182/blood-2012-02-413336 . 23053574

[7] Onai N, Kurabayashi K, Hosoi-Amaike M, Toyama-Sorimachi N, Matsushima K, Inaba K, et al A clonogenic progenitor with prominent plasmacytoid dendritic cell developmental potential. Immunity. 2013;38(5):943–57. Epub 2013/04/30. 10.1016/j.immuni.2013.04.006 . 23623382

[8] Chen YL, Chen TT, Pai LM, Wesoly J, Bluyssen HA, Lee CK. A type I IFN-Flt3 ligand axis augments plasmacytoid dendritic cell development from common lymphoid progenitors. J Exp Med. 2013;210(12):2515–22. Epub 2013/10/21. 10.1084/jem.20130536 . 24145513 PMC3832917

[9] Schmid MA, Kingston D, Boddupalli S, Manz MG. Instructive cytokine signals in dendritic cell lineage commitment. Immunol Rev. 2010;234(1):32–44. Epub 2010/03/03. 10.1111/j.0105-2896.2009.00877.x . 20193010

[10] Watowich SS, Liu YJ. Mechanisms regulating dendritic cell specification and development. Immunol Rev. 2010;238(1):76–92. Epub 2010/10/26. 10.1111/j.1600-065X.2010.00949.x 20969586 PMC3039024

[11] D'Amico A, Wu L. The early progenitors of mouse dendritic cells and plasmacytoid predendritic cells are within the bone marrow hemopoietic precursors expressing Flt3. J Exp Med. 2003;198(2):293–303. Epub 2003/07/23. 10.1084/jem.20030107 12874262 PMC2194069

[12] Karsunky H, Merad M, Cozzio A, Weissman IL, Manz MG. Flt3 ligand regulates dendritic cell development from Flt3+ lymphoid and myeloid-committed progenitors to Flt3+ dendritic cells in vivo. J Exp Med. 2003;198(2):305–13. Epub 2003/07/23. 10.1084/jem.20030323 12874263 PMC2194067

[13] McKenna HJ, Stocking KL, Miller RE, Brasel K, De Smedt T, Maraskovsky E, et al Mice lacking flt3 ligand have deficient hematopoiesis affecting hematopoietic progenitor cells, dendritic cells, and natural killer cells. Blood. 2000;95(11):3489–97. Epub 2000/05/29. . 10828034

[14] Waskow C, Liu K, Darrasse-Jeze G, Guermonprez P, Ginhoux F, Merad M, et al The receptor tyrosine kinase Flt3 is required for dendritic cell development in peripheral lymphoid tissues. Nature immunology. 2008;9(6):676–83. Epub 2008/05/13. 10.1038/ni.1615 18469816 PMC2746085

[15] Cisse B, Caton ML, Lehner M, Maeda T, Scheu S, Locksley R, et al Transcription factor E2-2 is an essential and specific regulator of plasmacytoid dendritic cell development. Cell. 2008;135(1):37–48. Epub 2008/10/16. 10.1016/j.cell.2008.09.016 18854153 PMC2631034

[16] Nagasawa M, Schmidlin H, Hazekamp MG, Schotte R, Blom B. Development of human plasmacytoid dendritic cells depends on the combined action of the basic helix-loop-helix factor E2-2 and the Ets factor Spi-B. Eur J Immunol. 2008;38(9):2389–400. Epub 2008/09/16. 10.1002/eji.200838470 . 18792017

[17] Ghosh HS, Cisse B, Bunin A, Lewis KL, Reizis B. Continuous expression of the transcription factor e2-2 maintains the cell fate of mature plasmacytoid dendritic cells. Immunity. 2010;33(6):905–16. Epub 2010/12/15. 10.1016/j.immuni.2010.11.023 21145760 PMC3010277

[18] Bao M, Liu YJ. Regulation of TLR7/9 signaling in plasmacytoid dendritic cells. Protein & cell. 2013;4(1):40–52. Epub 2012/11/08. 10.1007/s13238-012-2104-8 ; PubMed Central PMCID: PMCPmc3667388. 23132256 PMC3667388

[19] Chen LS, Wei PC, Liu T, Kao CH, Pai LM, Lee CK. STAT2 hypomorphic mutant mice display impaired dendritic cell development and antiviral response. J Biomed Sci. 2009;16:22 Epub 2009/03/11. 10.1186/1423-0127-16-22 19272190 PMC2653529

[20] Swiecki M, Gilfillan S, Vermi W, Wang Y, Colonna M. Plasmacytoid dendritic cell ablation impacts early interferon responses and antiviral NK and CD8(+) T cell accrual. Immunity. 2010;33(6):955–66. Epub 2010/12/07. 10.1016/j.immuni.2010.11.020 ; PubMed Central PMCID: PMCPmc3588567. 21130004 PMC3588567

[21] Takagi H, Fukaya T, Eizumi K, Sato Y, Sato K, Shibazaki A, et al Plasmacytoid dendritic cells are crucial for the initiation of inflammation and T cell immunity in vivo. Immunity. 2011;35(6):958–71. Epub 2011/12/20. 10.1016/j.immuni.2011.10.014 . 22177923

[22] Wan H, Dupasquier M. Dendritic cells in vivo and in vitro. Cellular & molecular immunology. 2005;2(1):28–35. Epub 2005/10/11. . 16212908

[23] O'Neill HC, Wilson HL. Limitations with in vitro production of dendritic cells using cytokines. J Leukoc Biol. 2004;75(4):600–3. Epub 2004/01/06. 10.1189/jlb.0903446 . 14704369

[24] Esashi E, Wang YH, Perng O, Qin XF, Liu YJ, Watowich SS. The signal transducer STAT5 inhibits plasmacytoid dendritic cell development by suppressing transcription factor IRF8. Immunity. 2008;28(4):509–20. Epub 2008/03/18. 10.1016/j.immuni.2008.02.013 18342552 PMC2864148

[25] Li HS, Yang CY, Nallaparaju KC, Zhang H, Liu YJ, Goldrath AW, et al The signal transducers STAT5 and STAT3 control expression of Id2 and E2-2 during dendritic cell development. Blood. 2012;120(22):4363–73. Epub 2012/10/04. 10.1182/blood-2012-07-441311 23033267 PMC3507145

[26] Naik SH, Proietto AI, Wilson NS, Dakic A, Schnorrer P, Fuchsberger M, et al Cutting edge: generation of splenic CD8+ and CD8- dendritic cell equivalents in Fms-like tyrosine kinase 3 ligand bone marrow cultures. J Immunol. 2005;174(11):6592–7. Epub 2005/05/21. 10.4049/jimmunol.174.11.6592 . 15905497

[27] Naik SH, Sathe P, Park HY, Metcalf D, Proietto AI, Dakic A, et al Development of plasmacytoid and conventional dendritic cell subtypes from single precursor cells derived in vitro and in vivo. Nature immunology. 2007;8(11):1217–26. Epub 2007/10/09. 10.1038/ni1522 . 17922015

[28] Onai N, Obata-Onai A, Schmid MA, Ohteki T, Jarrossay D, Manz MG. Identification of clonogenic common Flt3+M-CSFR+ plasmacytoid and conventional dendritic cell progenitors in mouse bone marrow. Nature immunology. 2007;8(11):1207–16. Epub 2007/10/09. 10.1038/ni1518 . 17922016

[29] Kondo M, Weissman IL, Akashi K. Identification of clonogenic common lymphoid progenitors in mouse bone marrow. Cell. 1997;91(5):661–72. Epub 1997/12/11. . 9393859 10.1016/s0092-8674(00)80453-5

[30] Onai N, Obata-Onai A, Tussiwand R, Lanzavecchia A, Manz MG. Activation of the Flt3 signal transduction cascade rescues and enhances type I interferon-producing and dendritic cell development. J Exp Med. 2006;203(1):227–38. Epub 2006/01/19. 10.1084/jem.20051645 16418395 PMC2118073

[31] Whitlock CA, Tidmarsh GF, Muller-Sieburg C, Weissman IL. Bone marrow stromal cell lines with lymphopoietic activity express high levels of a pre-B neoplasia-associated molecule. Cell. 1987;48(6):1009–21. Epub 1987/03/27. 10.1016/0092-8674(87)90709-4 . 3493849

[32] Chou WC, Levy DE, Lee CK. STAT3 positively regulates an early step in B-cell development. Blood. 2006;108(9):3005–11. Epub 2006/07/11. 10.1182/blood-2006-05-024430 16825489 PMC1895520

[33] Wang WB, Levy DE, Lee CK. STAT3 negatively regulates type I IFN-mediated antiviral response. J Immunol. 2011;187(5):2578–85. Epub 2011/08/04. 10.4049/jimmunol.1004128 . 21810606

[34] Bock TA. Assay systems for hematopoietic stem and progenitor cells. Stem Cells. 1997;15 Suppl 1:185–95. Epub 1997/01/01. 10.1002/stem.5530150824 . 9368340

[35] Szilvassy SJ, Weller KP, Lin W, Sharma AK, Ho AS, Tsukamoto A, et al Leukemia inhibitory factor upregulates cytokine expression by a murine stromal cell line enabling the maintenance of highly enriched competitive repopulating stem cells. Blood. 1996;87(11):4618–28. Epub 1996/06/01. doi: doi:None. . 8639830

[36] Arcanjo K, Belo G, Folco C, Werneck CC, Borojevic R, Silva LC. Biochemical characterization of heparan sulfate derived from murine hemopoietic stromal cell lines: a bone marrow-derived cell line S17 and a fetal liver-derived cell line AFT024. J Cell Biochem. 2002;87(2):160–72. Epub 2002/09/24. 10.1002/jcb.10293 . 12244569

[37] Fancke B, Suter M, Hochrein H, O'Keeffe M. M-CSF: a novel plasmacytoid and conventional dendritic cell poietin. Blood. 2008;111(1):150–9. Epub 2007/10/06. 10.1182/blood-2007-05-089292 . 17916748

[38] Stanley ER. Lineage commitment: cytokines instruct, at last! Cell stem cell. 2009;5(3):234–6. Epub 2009/09/08. 10.1016/j.stem.2009.08.015 ; PubMed Central PMCID: PMCPmc4049006. 19733531 PMC4049006

[39] Kingston D, Schmid MA, Onai N, Obata-Onai A, Baumjohann D, Manz MG. The concerted action of GM-CSF and Flt3-ligand on in vivo dendritic cell homeostasis. Blood. 2009;114(4):835–43. Epub 2009/05/26. 10.1182/blood-2009-02-206318 . 19465690

[40] Essers MA, Offner S, Blanco-Bose WE, Waibler Z, Kalinke U, Duchosal MA, et al IFNalpha activates dormant haematopoietic stem cells in vivo. Nature. 2009;458(7240):904–8. Epub 2009/02/13. 10.1038/nature07815 . 19212321

[41] Contractor N, Louten J, Kim L, Biron CA, Kelsall BL. Cutting edge: Peyer's patch plasmacytoid dendritic cells (pDCs) produce low levels of type I interferons: possible role for IL-10, TGFbeta, and prostaglandin E2 in conditioning a unique mucosal pDC phenotype. J Immunol. 2007;179(5):2690–4. Epub 2007/08/22. . 17709480 10.4049/jimmunol.179.5.2690

[42] Banchereau J, Pascual V. Type I interferon in systemic lupus erythematosus and other autoimmune diseases. Immunity. 2006;25(3):383–92. Epub 2006/09/19. 10.1016/j.immuni.2006.08.010 . 16979570

