# Supplementary figures and images for: Efficient Generation of Plasmacytoid Dendritic Cell from Common Lymphoid Progenitors by Flt3 Ligand
Source: PLoS One. 2015 Aug 11;10(8):e0135217. doi: 10.1371/journal.pone.0135217 (PMC4532451; doi:10.1371/journal.pone.0135217)

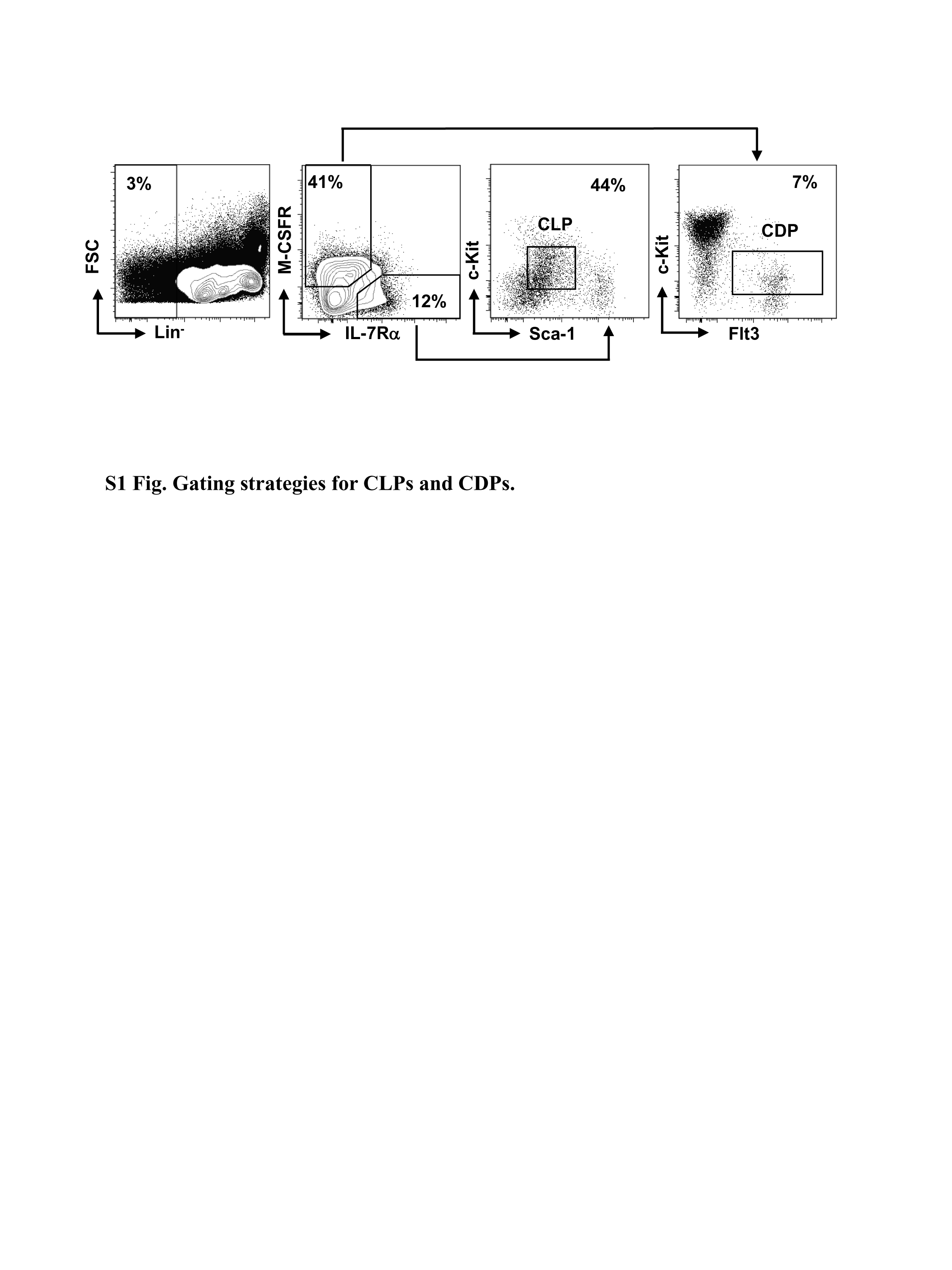

Supplement: S1 Fig — CLPs were defined as lin-c-kitint Sca-1intM-CSFR-IL7Ra+. CDPs were defined as lin-c-kitint Flt3+M-CSFR+IL-7Ra-. (TIF) [file pone.0135217.s001.tif]

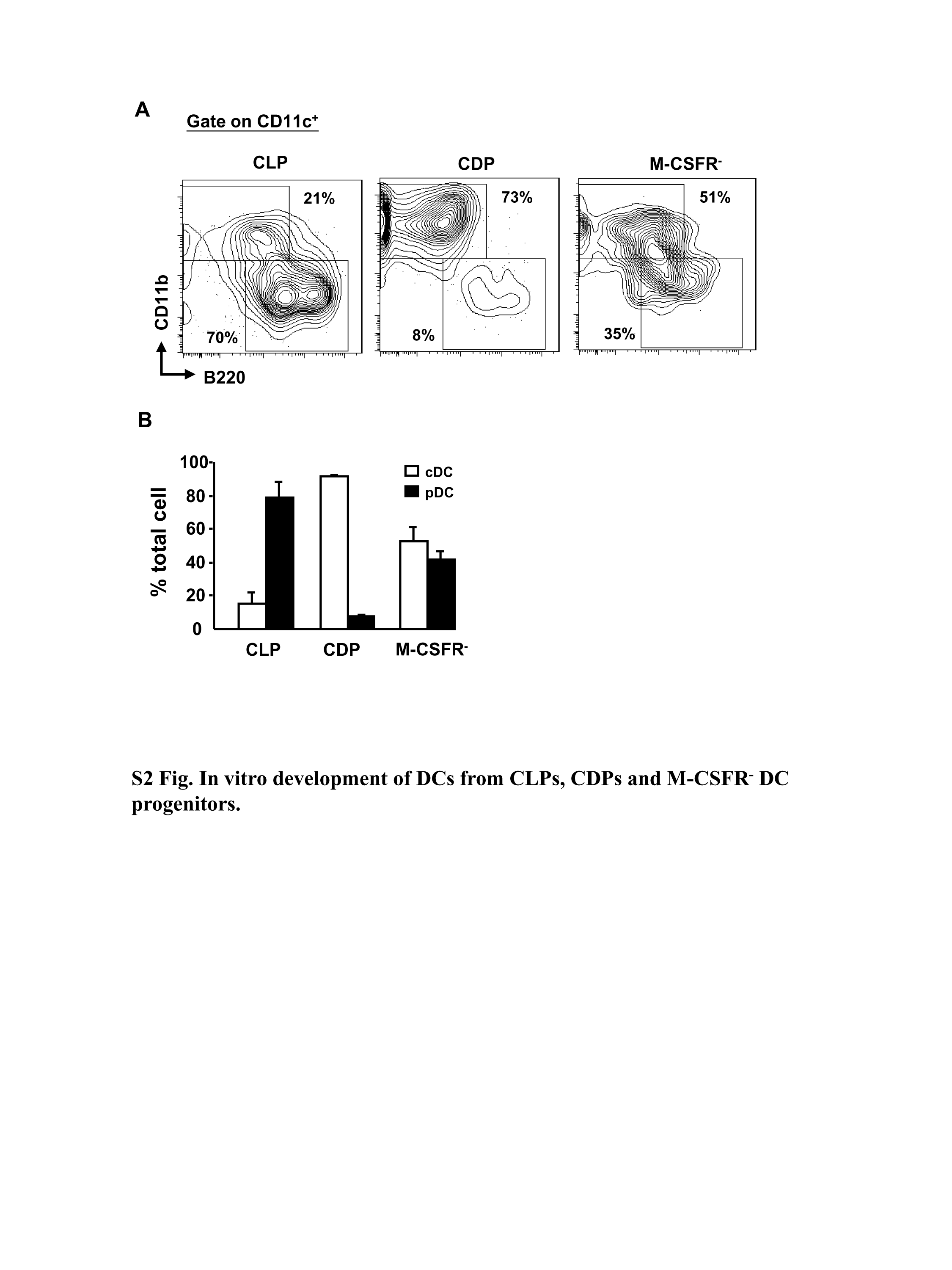

Supplement: S2 Fig — (A) CLPs, CDPs, and M-CSFR- DC progenitors (Lin-c-Kitint/loFlt3+ M-CSFR-) were sorted and cultured in vitro under feeder-free conditions in the presence of FL (100 ng/ml) for 6 d. The progeny cells were stained, gated on CD11c+ and analyzed by flow cytometry. (B) Mean percentages of cDC (CD11c+CD11b+ B220-) and pDC (CD11c+CD11b-B220+) are shown (n = 4). (TIF) [file pone.0135217.s002.tif]

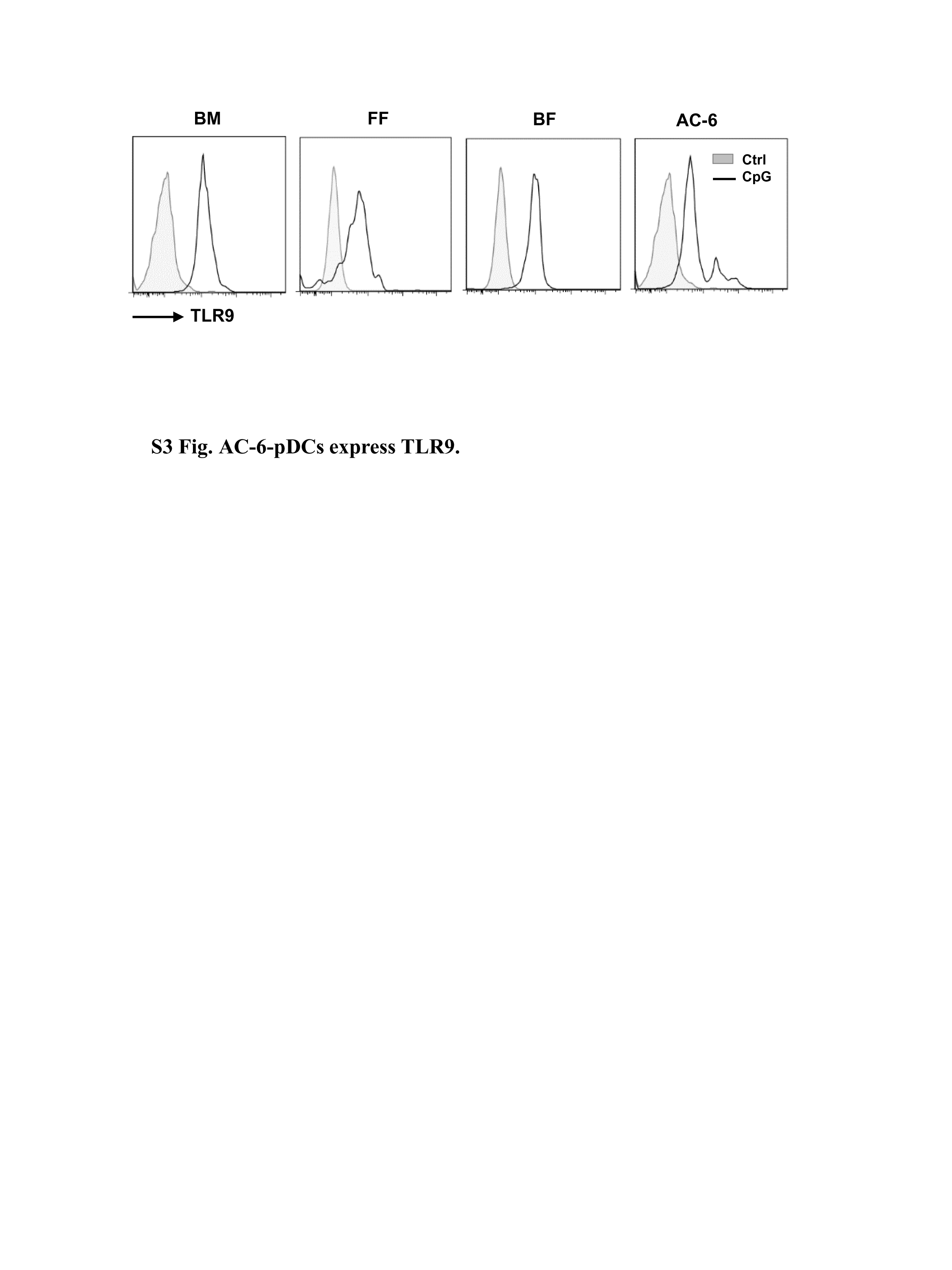

Supplement: S3 Fig — pDCs sorted from BM or BF system or DCs derived from FF and AC-6 system were treated with or without CpG ODN (1 μg/ml) for 24 h. Expressions of TLR9 on sorted or gated pDCs are shown. (TIF) [file pone.0135217.s003.tif]

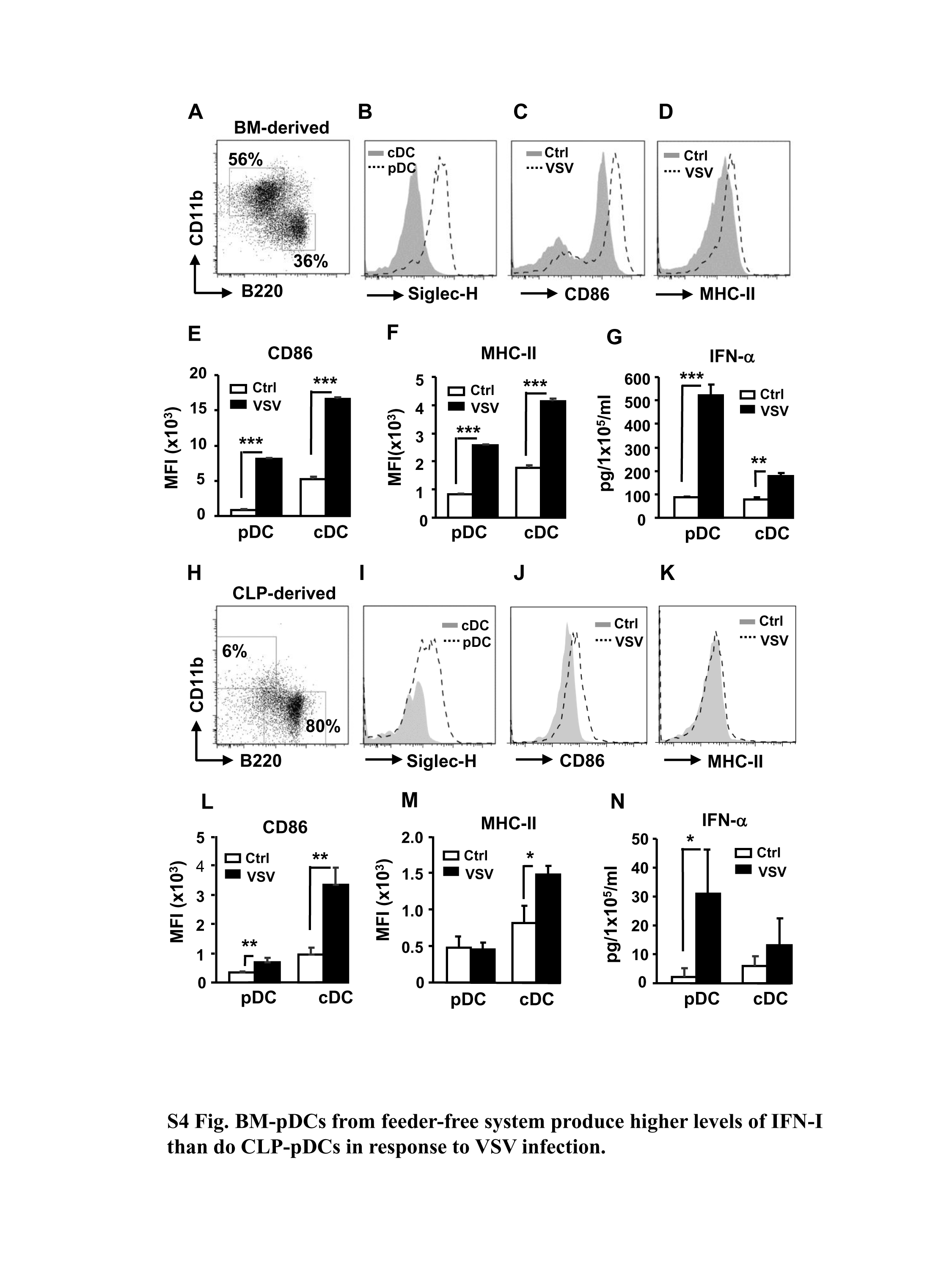

Supplement: S4 Fig — BM or CLPs of WT mice were cultured in vitro in the presence of FL 100 ng/ml for 6 d. BM (A-G) or CLP (H-N)-derived cells were stained with anti-CD11c, anti-CD11b, anti-B220 and anti-Siglec-H, gated on CD11c+ cells and analyzed for cDCs (CD11c+CD11b+B220-) and pDCs (CD11c+CD11b-B220+). The expression of Siglec-H on cDCs and pDCs from BM (B) or CLPs (I) is shown. Magnetic bead-purified pDCs from BM-derived DCs and CLP-derived DCs were infected with VSV at an MOI = 10 for 24 h. Infected pDCs were stained for anti-CD86 (C, E, J, L) and anti-MHC-II (D, F, K, M). (n = 3–4) The supernatant of the infected BM-derived pDCs (G) or CLP-derived pDCs (N) was subjected to ELISA for measuring secreted IFN-α. (n = 3), *P<0.05, **P<0.01, ***P<0.005. (TIF) [file pone.0135217.s004.tif]

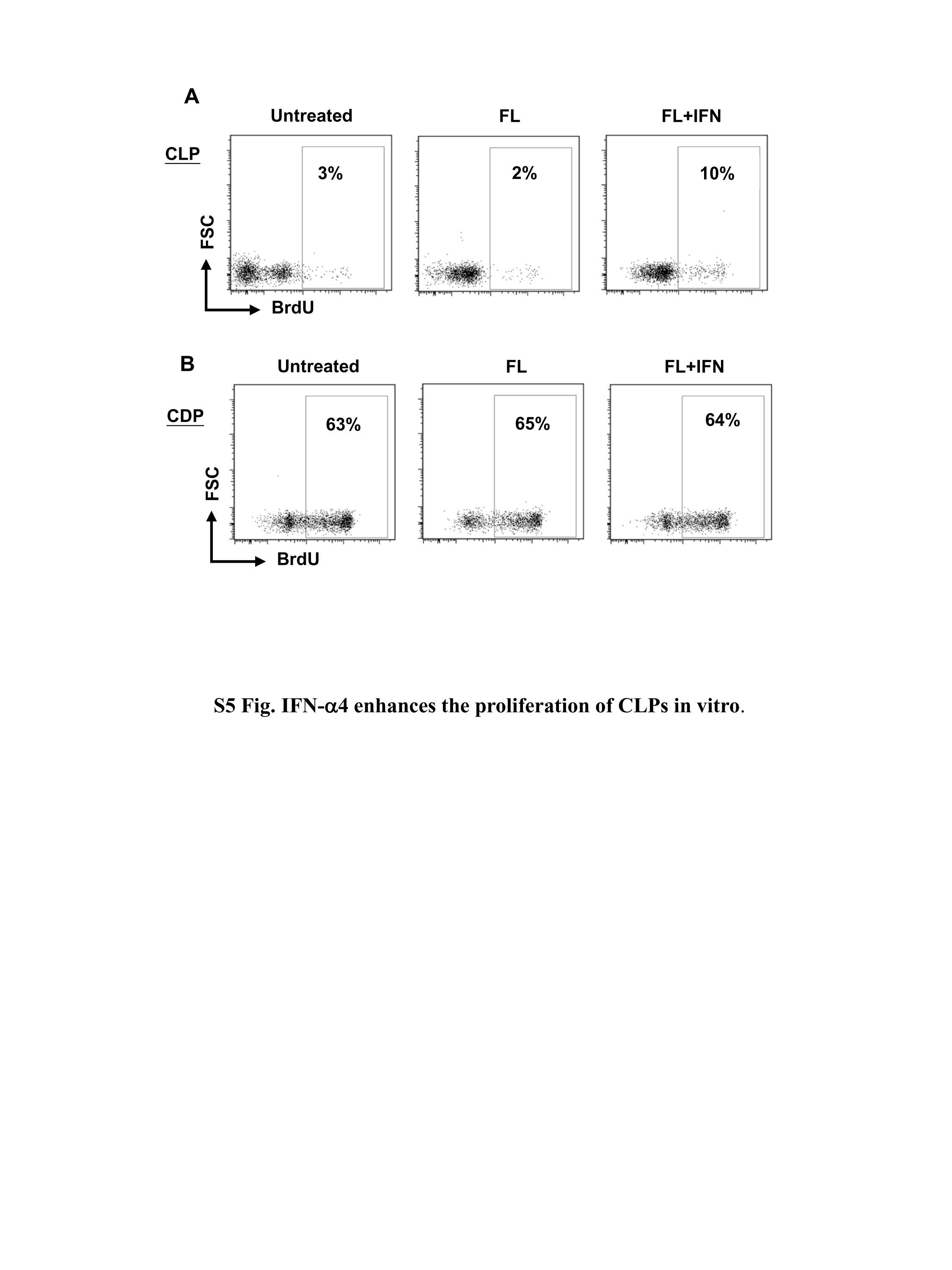

Supplement: S5 Fig — CLPs (A) and CDPs (B) sorted from WT mice were stimulated with or without FL alone (100 ng/ml) or FL (100 ng/ml) plus IFN-α4 (100 U/ml) for 8 h. BrdU (100 μg/ml) was added at the last 4 h before subjecting the treated cells to intracellular staining for BrdU as described in the Materials and Methods. (TIF) [file pone.0135217.s005.tif]
